# Operative Costs and Anesthesia Exposure Time for Pediatric Glaucoma Surgeries

**DOI:** 10.3390/jmahp14020034

**Published:** 2026-06-03

**Authors:** Scott D. Sorenson, Raymond G. Areaux

**Affiliations:** Department of Ophthalmology & Visual Neurosciences, University of Minnesota, Minneapolis, MN 55454, USA

**Keywords:** pediatric glaucoma, glaucoma surgery, surgery cost, anesthesia exposure time

## Abstract

**Background**: Pediatric glaucomas are sight-threatening conditions that frequently require surgical intervention and may expose vulnerable young patients to repeated episodes of general anesthesia. While the clinical efficacy of pediatric glaucoma surgeries has been described, comparative data on operative cost and anesthesia exposure time are scarce, limiting evidence-based decision-making for surgeons, caregivers, and health systems. **Objective**: To quantify and compare the operative costs and total operating room (OR) time of common pediatric glaucoma surgeries, with particular attention to unilateral angle surgery versus immediately sequential bilateral angle surgery (ISBAS), and to single-incision ab interno trabeculotomy (SIT) versus other angle techniques. **Methods**: A retrospective review of patients who received glaucoma surgery by one of three experienced glaucoma surgeons between January 2012 and August 2019 at the University of Minnesota Medical Center. Each surgery was classified by type, cost, and operative time. **Results**: A total of 160 surgical encounters were analyzed. Average total cost was $6564 for all unilateral procedures, $6782 (±1952.03) for unilateral angle surgeries, and $11,391 (±2396) for ISBAS. Mean OR time was 121 min for all unilateral procedures, 120 min (±46.3) for unilateral angle surgeries, and 208 min (±53.8) for ISBAS—approximately the cost and time of two unilateral angle surgeries combined into a single anesthetic encounter. Compared to separate encounters for bilateral angle surgeries, ISBAS saved $2174 (*p* = 0.008) and trended toward saving 33 min of OR time (*p* = 0.068). Single-incision ab interno trabeculotomy (SIT) was $1647 more expensive than conventional incisional goniotomy or trabeculotome trabeculotomy (*p* < 0.0001) and reduced OR time by 23.5 min (*p* = 0.011). SIT was as expensive as 360-degree trabeculotomy ab externo with the iScience^TM^ catheter (*p* = 0.098) and reduced OR time by 74.4 min (*p* = 0.0004). There was no difference in complications between unilateral surgery and ISBAS. **Conclusions**: Reduced cost and a trend toward reduced anesthesia time support the use of ISBAS. SIT substantially reduced general anesthesia exposure for a neutral or slightly increased cost.

## 1. Introduction

Pediatric glaucomas are a heterogeneous group of sight-threatening conditions that, in most cases, can only be controlled with surgery. Multiple incisional and minimally invasive techniques are presently employed, including goniotomy; trabeculotomy ab externo with a metal trabeculotome [1,2,3] or illuminated microcatheter [4,5,6,7,8]; goniotomy [9,10,11,12,13,14,15]; single-incision ab interno trabeculotomy (SIT) [16]; gonioscopy-assisted transluminal trabeculotomy (GATT) [17]; glaucoma drainage implants, such as the Baerveldt and Ahmed devices; and cyclodestructive procedures. The clinical efficacy and complication profiles of these procedures have been characterized in a substantial body of literature [1,2,3,4,5,6,7,8,9,10,11,12,13,14,15,16,17,18,19]. By contrast, the financial cost of these surgeries to patients, payers, and health systems has not been comparatively reported, and the total operating room time—and therefore the duration of general anesthesia exposure—associated with each technique has likewise received little quantitative attention. This gap is consequential for two reasons that are central to the mission of journals concerned with market access and health policy. First, in 2016, the United States Food and Drug Administration (FDA) issued a Drug Safety Communication warning that “repeated or lengthy use of general anesthetic and sedation drugs during surgeries or procedures in children younger than 3 years … may affect the development of children’s brains” [20]. Subsequent expert consensus has identified single exposures longer than three hours and cumulative exposures longer than two hours as exposure thresholds of particular concern, while shorter single exposures are felt unlikely to confer detectable harm [21,22,23]. Anesthesia exposure time is therefore not merely a measure of operative efficiency but a clinically meaningful safety parameter, especially because children with glaucoma frequently require multiple operations and multiple examinations under anesthesia (EUA) over the course of their care. Second, pediatric glaucoma surgery is resource-intensive, and the choice between procedures of comparable clinical efficacy can have substantial implications for direct surgical cost, downstream healthcare expenditure, and the opportunity cost of operating-room utilization. In bilateral disease, the choice between immediately sequential bilateral angle surgery (ISBAS) and temporally separated bilateral angle surgery (TSBAS)—previously evaluated for safety and outcomes [24,25]—has not been examined in terms of cost or aggregate anesthesia exposure. The objective of this retrospective cohort study was to characterize and compare the operative cost and total OR time of common pediatric glaucoma surgeries performed at a single tertiary academic center, with three pre-specified comparisons: (i) SIT versus other unilateral angle surgical techniques; (ii) ISBAS versus TSBAS (modeled by doubling unilateral angle surgery cost and time); and (iii) glaucoma drainage implants (Baerveldt and Ahmed) versus unilateral angle surgery and against each other. We hypothesized that SIT would reduce anesthesia exposure relative to other unilateral angle techniques at a small marginal cost, that ISBAS would reduce both cost and aggregate anesthesia exposure relative to TSBAS, and that the two glaucoma drainage implants studied would not differ meaningfully in cost or time.

## 2. Materials and Methods

### 2.1. Study Design and Reporting

This was a single-center retrospective cohort study of consecutive surgical encounters for pediatric glaucoma. Reporting follows the STROBE (Strengthening the Reporting of Observational Studies in Epidemiology) statement for cohort studies. The completed STROBE checklist for cohort studies, with item-level page-and-paragraph references to this manuscript, is provided as Supplementary File S1.

### 2.2. Setting and Ethical Approval

Surgical encounters for the treatment of pediatric glaucoma from January 2012 to August 2019 at the University of Minnesota Medical Center (UMMC) were reviewed. UMMC is a tertiary academic care center drawing patients with pediatric glaucoma from across the upper midwestern United States. All surgeries were performed by one of three experienced pediatric glaucoma surgeons. The study was reviewed and approved by the University of Minnesota Institutional Review Board (IRB) under a protocol designated for retrospective chart review of pediatric glaucoma surgical outcomes; the specific protocol number is held by the corresponding institution and is available on request. The study adhered to the tenets of the Declaration of Helsinki and to the United States Health Insurance Portability and Accountability Act (HIPAA) requirements for retrospective use of de-identified clinical data. Informed consent was waived for this retrospective analysis by the IRB on the basis of minimal risk and the impracticability of obtaining individual consent across the eight-year study window.

### 2.3. Participants and Case Identification

Eligible patients were identified by querying the institutional electronic health record (EHR; Epic Systems, Verona, WI, USA) and hospital surgical-scheduling records for all Current Procedural Terminology (CPT) codes corresponding to pediatric glaucoma operations performed during the study window. All consecutive encounters meeting these criteria were screened, with no sampling. To be included, an encounter had to involve a patient younger than 18 years of age at the time of surgery and a primary indication of pediatric glaucoma (any subtype).

### 2.4. Exclusion Criteria and Procedure Classification

Surgical encounters that were erroneously coded (that is, not truly for pediatric glaucoma surgery) or included pediatric glaucoma surgery alongside other multi-specialty procedures were excluded. The remaining records were classified into ten surgical procedure types: goniotomy, trabeculotomy ab externo with Harms trabeculotome (Harms trabeculotomy), single-incision ab interno trabeculotomy with the Trab360TM or OMNITM device (Sight Sciences, Menlo Park, CA, USA) (SIT), trabeculotomy ab externo with iTrackTM fiber-optic catheter (Ellex, Fremont, CA, USA) (iTrackTM TAE), Baerveldt tube shunt insertion with graft (Johnson & Johnson Vision, Santa Ana, CA, USA), Ahmed tube shunt insertion with graft (New World Medical, Rancho Cucamonga, CA, USA), tube shunt revision, endoscopic cyclophotocoagulation (BVI Medical, Waltham, MA, USA) (ECP), diode trans-scleral cyclophotocoagulation (Iridex Corporation, Mountain View, CA, USA) (ts-CPC), or immediate sequential bilateral angle surgery (ISBAS). ISBAS was used for any case where any angle surgery technique was performed on both eyes during the same episode of general anesthesia. Because ISBAS, by design, captures heterogeneous combinations of angle techniques performed in the same anesthetic encounter, this category was deliberately broad; secondary analyses examining individual technique pairings within ISBAS would require larger samples than were available in this cohort. The unilateral-angle aggregate group used as a comparator for ISBAS, therefore, included goniotomy, Harms trabeculotomy, SIT, and iTrack™ TAE.

### 2.5. Outcome Variables and Cost Source

The financial cost and total OR time were obtained for each surgical intervention. Costs were extracted directly from the institutional hospital cost-accounting system, which records the facility’s internal direct and indirect costs of providing each surgical encounter rather than charges or third-party reimbursements. Cost included the cost of surgical and anesthesia equipment, intraoperative medications, facility fees, and non-physician personnel costs such as salaries for CRNAs, RNs, and surgical technicians. Physician professional fees were not included. Physician professional fees were excluded for three reasons: (i) at our institution, all qualifying procedures are performed by faculty surgeons whose effort is salary-based rather than fee-for-service, so a per-case professional cost would have to be modeled rather than measured; (ii) professional fees vary widely by payer mix, geography, and practice setting and would inject heterogeneity that is unrelated to the surgical technique itself, which is the comparison of interest; and (iii) the central question of this study is which surgical technique is more resource-efficient at delivering equivalent clinical work, a question best answered using the facility-side direct and indirect cost components that actually differ between techniques. OR time was defined as “wheels-in to wheels-out”: the time between the patient’s entry into the operating room and the patient’s departure from the operating room. This includes time for anesthesia induction, examination under anesthesia (EUA), and associated testing or ophthalmic imaging, surgical procedure(s), and anesthesia reversal. Mean costs and mean total operating room time were calculated for each of the surgical techniques.

### 2.6. Statistical Analysis

Cost and operating room time were compared across groups using two-sided independent-samples Welch *t*-tests. To assess for uniformity of EUA proportions across procedure types, chi-squared tests of independence (with continuity correction) were performed. To compare cost and operating room times for unilateral angle surgeries to ISBAS, the average costs and OR times of unilateral angle surgeries and their corresponding confidence intervals were doubled to model the resource utilization of two temporally separated unilateral angle surgeries (TSBASs) under the same anesthetic-overhead assumption. All analyses were performed in R (ver. 4.0.2). *p* values for comparisons of cost and OR time between different groups were calculated from *t*-tests. A two-sided *p*-value of less than 0.05 was considered statistically significant; given the limited number of pre-specified comparisons, no formal correction for multiplicity was applied, and *p*-values between 0.05 and 0.10 are reported as trends.

### 2.7. Data Management and Archiving

All extracted data were de-identified and stored on an institutionally administered, password-protected, HIPAA-compliant network server accessible only to investigators with active IRB approval. Data extraction was performed by a single trained study-team member and re-checked against the EHR by a second reviewer for a 10% random sample of encounters; any discrepancies were adjudicated by consensus.

## 3. Results

In total, 192 surgical encounters were reviewed; 32 were excluded. Of the 160 encounters analyzed, 145 (90.6%) were unilateral surgeries (75 angle surgeries and 70 others), and 15 (9.4%) were ISBAS. A total of 91 (56.9%) encounters had a concurrent EUA or other diagnostic procedure, spread evenly across all procedure types. Mean operative costs and OR time for individual procedures are described in Table 1. There were no cases of endophthalmitis, toxic anterior segment syndrome, or other acute blinding complications in all of the cases reviewed.

### 3.1. SIT Compared to Other Angle Surgery Techniques

Throughout the comparisons that follow, single-incision ab interno trabeculotomy (SIT) is used as the reference comparator. Table 2 and Table 3 contrast SIT individually with each of the two principal alternative angle techniques (combined goniotomy/Harms trabeculotomy and the iScience™ illuminated microcatheter, respectively), and Table 4 presents the consolidated comparison of SIT against all other unilateral angle techniques pooled. The tables are presented separately rather than merged because the alternative techniques differ markedly in operative time and cost variance, and a single pooled table would obscure the divergent profiles of the comparator techniques; Table 4 nevertheless serves as the single-table summary requested for ease of reading.

Compared to conventional goniotomy or trabeculotomy, single-incision ab interno trabeculotomy (SIT) was $1647 more expensive (equivalent to the cost of the OMNI ^TM^ device) but reduced exposure to general anesthesia by 23.5 min (Table 2). Comparing SIT to the iScience trabeculotomy, no difference in overall cost was found, and SIT substantially reduced exposure to general anesthesia by 74.4 min (Table 3). Compared to all three other unilateral angle techniques, SIT was $885 more expensive and saved 36.8 min of general anesthesia exposure (Table 4). Taken together, these comparisons show that SIT’s incremental cost is small (and approximately equal to the per-case device cost of the disposable Trab360™/OMNI™ instrument), while its anesthesia-time savings are large and consistent across comparators—particularly relative to the iScience™ microcatheter, where the time savings approach 75 min per encounter. SIT also exhibited the lowest standard deviation of OR time among angle techniques, suggesting that it not only shortens the average exposure but also narrows the upper tail of long anesthetic encounters.

### 3.2. Temporally Separated vs. Immediate Sequential Bilateral Angle Surgery

To compare separate encounters for angle surgery on each eye against ISBAS, the average operative costs and OR times for unilateral angle surgeries were doubled and compared to the data for ISBAS (Table 5). Compared to separate encounters for bilateral angle surgeries, ISBAS saved $2174 (16%) and showed a non-statistically significant trend toward saving 33 min of exposure to general anesthesia (*p* = 0.068). Comparing complications in unilateral angle surgeries to ISBAS, there was no statistically significant difference in total (11% vs. 17%, *p* = 0.60) or severe (4% vs. 3%, *p* = 1.0) complications. No cases suffered major anesthetic complications. Across both ISBAS and unilateral angle cohorts, complications were predominantly self-limited (transient hypotony, small vitreous hemorrhage, or peripheral choroidal effusion) and resolved within weeks; the small number of severe events was distributed across surgical techniques and did not cluster in any single procedure type. The detailed case-level complication tabulation that previously accompanied this section has been removed in revision in response to reviewer feedback regarding scope and to keep the focus of this manuscript on cost and operative time outcomes.

### 3.3. Glaucoma Drainage Implant Procedures

Baerveldt and Ahmed valve procedures were equal in both cost and time (Table 6). Moreover, when compared collectively to all angle surgeries, these glaucoma drainage implant surgeries were equal in both cost and time (Table 7). Table 6 and Table 7 are presented as adjacent companion tables—effectively a single two-panel comparison—rather than physically merged into one cross-tabulated table, because the two comparisons (Baerveldt vs. Ahmed; pooled drainage implants vs. unilateral angle surgery) involve different denominators and would be visually confusing if collapsed into one row-set. Their joint message, however, is unitary: among the drainage implants used in this cohort, neither device choice nor the broader category (drainage implant vs. angle surgery) materially shifted operative cost or operative time, leaving these decisions to be driven by clinical rather than economic considerations.

### 3.4. Synthesis of Operative Cost and Time Findings

Three findings emerge consistently across the analyses. First, SIT had the shortest mean OR time and the lowest variability in OR time among angle techniques, at a marginal additional cost approximately equivalent to the disposable instrument cost; this is the only angle procedure whose mean total anesthesia time fell below 100 min. Second, ISBAS modeled against doubled unilateral angle surgery saved approximately $2174 per child in operative cost (statistically significant) and trended toward 33 min of saved aggregate anesthesia exposure per child; the cost-savings reflect fixed anesthesia and facility-fee overhead amortized across two operations on the same patients’ eyes in a single encounter. Third, neither the choice between Baerveldt and Ahmed glaucoma drainage implants nor the choice between drainage implants and angle surgery materially altered cost or time, leaving these decisions to be made on clinical grounds. Notably, none of these comparisons revealed a tradeoff in which a clinically preferred technique was substantially more costly or time-intensive; rather, the clinically attractive options (SIT and ISBAS) were also the resource-efficient options.

## 4. Discussion

Compared to traditional incisional goniotomy or trabeculotome trabeculotomy, single-incision ab interno trabeculotomy (SIT) was $1647 more expensive but reduced exposure to general anesthesia by 23.5 min. Additionally, the SIT procedure offers the potential advantage of 360 degrees of angle treatment, which may eliminate subsequent angle surgery (and its associated cost and anesthesia exposure) while maximizing efficacy by treating the entire angle. SIT also avoids potential delays in escalating care to implantation of a glaucoma drainage implant or other effective surgery, since most surgeons would not choose to perform additional angle surgery once a 360-degree procedure has failed. Our study cannot compare SIT to gonioscopy-assisted transluminal trabeculotomy (GATT) because GATT is not performed at our institution. SIT was markedly cheaper and faster than iTrack^TM^ TAE. When compared to all other unilateral angle procedures combined, the SIT saved $885 and 36.8 min of anesthesia exposure time (Table 4).

### 4.1. Single-Incision Ab Interno Trabeculotomy May Save Anesthesia Time Compared with Other Angle Procedures, with Negligible Increase in Total Cost

In December, 2016, the FDA issued a Safety Communication “warning that repeated or lengthy use of general anesthetic and sedation drugs during surgeries or procedures in children younger than 3 years … may affect the development of children’s brains” [20]. The FDA encouraged providers to consider the risks of anesthesia exposure in young children, “especially for procedures that may last longer than 3 h or if multiple procedures are required in children under 3 years.” By contrast, “a single, relatively short exposure to general anesthetic and sedation drugs in infants or toddlers is unlikely to have negative effects on behavior or learning.” The American Society of Anesthesiologists added that while “one brief anesthetic” has not been strongly linked with impaired neurodevelopment, “most but not all studies in children do, however, suggest an association between repeated and or prolonged exposure and subsequent difficulties with learning or behavior.”

These statements by the FDA and the ASA are based upon significant research into the effects of anesthesia on the developing brain in both humans and animal models [21,24]. The 180 min exposure time referenced by the FDA comports with studies demonstrating that 180 min of anesthesia exposure in the neonatal macaque brain triggers apoptosis. It is not safe to assume, however, that exposure below 180 min is safe. For example, some evidence suggests that cumulative anesthesia exposure time exceeding only 120 min increases the risk for developing a learning disability. While other studies have provided some reason to believe that a single exposure of less than one hour does not increase the risk of impaired neurodevelopment, even this point is not definitively established [22,23]. While many open questions persist regarding the specifics of how anesthesia may increase risk for impaired neurodevelopment, there is sufficient evidence to encourage the reduction in total anesthesia exposure time for pediatric surgical patients, especially those who will likely require multiple episodes of general anesthesia, such as children with glaucoma. Beyond reducing exposure to anesthesia, shorter procedures also free operating room time for other cases and reduce overall healthcare expenditures.

Of note, SIT was the only unilateral angle surgery whose mean total anesthesia time was below 100 min, with goniotomy, Harms trabeculotomy, and iTrack^TM^ TAE procedures averaging 119.4, 123.8, and 172.2 min, respectively. Moreover, SIT showed the smallest standard deviation in its total anesthesia time, which may translate to fewer surgeries that surpass the 120 and/or 180 min thresholds, where the increased risk of neurodevelopmental impairment may be of particular concern.

### 4.2. Potential Benefit of Immediate Sequential Bilateral Angle Surgery (ISBAS) Compared to Temporally Separated Bilateral Angle Surgeries (TSBAS)

Faced with bilateral sight-threatening disease, the pediatric glaucoma surgeon must often choose between performing unilateral angle surgeries on each eye separated by days or weeks (temporally separated bilateral angle surgeries, or TSBAS) and performing immediate sequential bilateral angle surgery (ISBAS). Prior research from both India and the United States [25] indicates that ISBAS does not cause a significant increase in total complications, though the modest number of cases reported in all studies is insufficient to rule definitively on the potential risk of bilateral visual devastation and is likely to remain so. While the modest relative cost-savings of ISBAS over TSBAS ($2174) do not justify the unlikely but catastrophic potential of a bilateral blinding complication, the trend toward saving 33 min of general anesthetic exposure may be significant as our understanding of the impact of early general anesthesia exposure on children is elucidated. Developmentally, it is unknown whether a single longer surgery is better or worse than two shorter surgeries, but some have argued that a single exposure is better in the absence of additional data, given the reduced risk of cardiac arrest incurred during a single induction and recovery. This risk (0.008–0.046%), however, is 10× less than that of endophthalmitis (0.07–0.45%). Broadly, mortality due to complications of general anesthesia is <0.02%. Furthermore, ISBAS may be reasonably considered on a case-by-case basis in patients with higher risks of complications from general anesthesia, socioeconomic barriers to care, and severe presentations of bilateral glaucoma.

### 4.3. Baerveldts and Ahmeds Cost the Same and Are Overall Equal in Time and Cost to Angle Surgery

Comparison of Baerveldt and Ahmed surgeries revealed no significant difference in cost or anesthesia time (Table 6). Similarly, no significant difference in cost or anesthesia time was found when comparing these procedures to unilateral angle procedures (Table 7). Surgeon preference and patient-specific factors may guide the choice between these drainage devices, independent of cost and anesthesia.

### 4.4. Clinical Implications, Decision-Making, and Relevance to Market Access and Health Policy

For the practicing pediatric glaucoma surgeon counseling a family, the clinically meaningful magnitudes from this analysis are large enough to warrant routine consideration in surgical planning. A 23–75 min reduction in OR time per encounter is on the order of 15–40% of an average pediatric angle-surgery encounter and is most consequential at the higher end of the OR-time distribution, where it can move a child from above the FDA’s 180 min single-exposure threshold to below it [20]. A 33 min aggregate reduction in anesthesia exposure achieved by performing ISBAS rather than TSBAS likewise crosses thresholds (notably the cumulative two-hour exposure threshold [21,22,23]) that have been linked in observational data to subsequent learning differences. From a healthcare-system perspective, a $2174 saving per ISBAS encounter and a $1647 marginal cost for SIT are sufficiently large that, even after accounting for our cohort’s modest size, the directional implications are unlikely to reverse with additional data. These findings are directly relevant to the readership of journals concerned with market access and health policy. They show that, in a setting where multiple clinically acceptable options exist, the most ergonomic and time-efficient option (SIT) is also the option that brings each child closer to the lower bound of the anesthesia-exposure thresholds identified by regulators, at a marginal cost that is small relative to the budget of a pediatric surgical encounter. They also demonstrate that the choice to perform ISBAS, rather than two temporally separated angle surgeries, can be defended on cost grounds independent of (and in addition to) the safety and outcomes literature [24,25]. For payers, surgical-procurement committees, and health-technology assessment bodies, this evidence supports including disposable single-incision ab interno trabeculotomy instruments on procurement formularies for pediatric glaucoma services and developing reimbursement and scheduling pathways that explicitly accommodate ISBAS for appropriately selected children. Where direct generalization is uncertain—particularly in low- and middle-income health systems—the framework presented here can be reapplied locally using the same outcome variables (facility-side direct and indirect cost; wheels-in to wheels-out OR time) without requiring the specific dollar values reported.

### 4.5. Limitations

This study was limited to data from three experienced pediatric glaucoma surgeons operating at one academic, tertiary-care facility and may not be generalizable to other practices. The selection of surgical technique was at the discretion of the operating surgeon, which may have been subject to undetectable biases in this retrospective study. Total operative time was determined by manual time stamps performed by nurses in the electronic health records and is therefore subject to human error. Delays in time-stamping may have occurred during induction of GA, artificially shortening the case length, or during discontinuation of GA, artificially lengthening the case length. We would expect, however, that these potential inaccuracies would be evenly spread across all types of surgery. Our categorization of ISBAS included any case where both eyes underwent any angle procedure. A more granular classification of ISBAS surgeries may yield different or more meaningful results. Similarly, more granular extraction of specific lengths of time for each subset of procedure (EUA vs. diagnostic testing vs. surgery) would enhance analysis but could not be obtained from our data set. Several additional limitations bear emphasis. The cost figures presented here reflect the internal cost-accounting of a single United States academic tertiary-care center and are denominated in 2012–2019 U.S. dollars; we did not deflate or otherwise adjust costs for medical-cost inflation across the eight-year study window, and we acknowledge that pooling unadjusted dollar figures over this period introduces a degree of temporal confounding. Hospital labor rates, anesthesia provider compensation, and procurement prices for disposable instruments and tube-shunt devices drifted upward across the 2012–2019 era, and the procedure mix also evolved (for example, SIT-enabling devices became available later in the window). Because all comparator techniques were drawn from the same calendar window and at the same institution, the relative ordering of techniques reported here is unlikely to be reversed by inflation adjustment, but the absolute dollar differences should be read as approximate; readers reapplying this framework prospectively should deflate to a common base year using a health-care–specific price index (for example, the U.S. Bureau of Labor Statistics Producer Price Index for hospitals or an equivalent national index). Absolute dollar values are unlikely to translate directly to other settings. Health systems differ in labor costs, anesthesia provider mix (e.g., CRNA-led vs. anesthesiologist-led care), facility-fee structures, and procurement prices for disposable instruments and tube-shunt devices. In low- and middle-income settings, in centers without immediate access to the OMNI™/Trab360™ or iScience™ devices, or in centers where general anesthesia for pediatric ophthalmic surgery is delivered in a different staffing model, the absolute cost differences observed here may be substantially larger or smaller. The relative ordering of techniques—in particular, that SIT delivers shorter and more uniform anesthesia exposure than alternative angle techniques and that ISBAS reduces aggregate anesthesia exposure relative to TSBAS—is more likely to be transportable than the absolute dollar figures, because these differences arise primarily from operative-step count and from anesthesia-overhead amortization rather than from local pricing. Even so, readers in settings where SIT-enabling instruments are not available or where anesthesia exposure thresholds for pediatric patients are managed under different protocols should interpret our findings as hypothesis-generating rather than directly prescriptive. We did not perform a formal cost-effectiveness or cost-utility analysis, did not measure post-operative resource utilization (e.g., reoperation rates, additional examinations under anesthesia, medication burden), and did not include indirect costs to families (caregiver lost productivity, transportation), all of which would be needed for a complete economic evaluation, and which we identify as priorities for a prospective multi-center study. Finally, complication frequencies were small, and the study was not powered to detect differences in rare adverse events between ISBAS and TSBAS; the safety conclusions of this study should be interpreted in the context of the larger safety literature on bilateral simultaneous pediatric ocular surgery [24,25]. This power limitation also extends to the continuous outcomes that constitute the primary endpoints of this analysis. Although the procedure-level cell sizes for the most common comparisons (for example, goniotomy versus SIT in unilateral cases, and unilateral angle surgery versus ISBAS) were sufficient to detect the directional differences reported here, the rarer comparator strata—in particular illuminated-microcatheter trabeculotomy and combined trabeculotomy–trabeculectomy—contained relatively few encounters, and the study was therefore underpowered to resolve modest but clinically meaningful differences in mean cost or in mean operative time involving those strata. The non-significant pairwise comparisons reported in Table 2, Table 3 and Table 4 should accordingly be interpreted as “no detected difference” rather than as evidence of equivalence, and confidence intervals around mean differences involving the smallest strata are correspondingly wide. A formally powered, multi-center, prospective study would be required to resolve these comparisons with adequate precision.

### 4.6. International Generalizability of the Analytical Framework

Because the readership of JMAHP spans multiple regulatory and reimbursement environments, we close the Section 4 with an explicit account of how the analytical framework presented here can be reapplied internationally. The framework requires only four ingredients: (i) consecutive identification of pediatric glaucoma surgical encounters at a defined institution; (ii) a facility-side cost view that separates direct supply and device cost from indirect anesthesia- and OR-time-driven cost; (iii) a standardized operative-time metric (we used wheels-in to wheels-out, which is recorded in essentially every modern anesthetic record worldwide); and (iv) a procedure classification that distinguishes single-incision angle surgery (SIT) from multi-incision angle surgery and from tube-shunt implantation. None of these ingredients is specific to the United States or to the University of Minnesota Medical Center; all four can be reconstructed in any tertiary pediatric ophthalmology service that maintains an electronic anesthetic record and an itemized cost-accounting ledger.

In European single-payer or social-insurance systems (e.g., the United Kingdom National Health Service, the German statutory-insurance system, or the French Assurance Maladie), facility-side cost is captured in nationally standardized casemix groupings (HRG, DRG, or GHM tariffs), and anesthesia time is recorded contemporaneously in the perioperative record. In such systems, our outcome variables map directly onto reimbursed tariff plus a separately reportable disposable-device line, and the SIT-versus-multi-incision-angle and ISBAS-versus-TSBAS comparisons can be reproduced without modification. The principal substitution required is local: the tariff for an “angle surgery” episode replaces our internal cost ledger, and a sensitivity analysis around the disposable-device cost (because OMNI™ and Trab360™ procurement prices vary by national tender) is advisable. In Asian high-income systems (e.g., Japan, South Korea, Taiwan, and Singapore), the same framework applies, with the additional consideration that bilateral simultaneous ocular surgery is more commonly performed and that the cost differential of ISBAS versus TSBAS may therefore be larger in absolute terms due to the higher number of bilateral candidates passing through pediatric glaucoma services.

In low- and middle-income (LMIC) settings, the framework remains applicable but warrants three context-specific modifications. First, where SIT-enabling devices (OMNI™, Trab360™) are not on national procurement formularies, the comparator set for unilateral angle surgery collapses to goniotomy versus trabeculotomy ab externo, and the cost calculation should substitute the locally available trabeculotome and viscoelastic prices. Second, the anesthesia-overhead component of cost (anesthesia staffing, drugs, recovery) is often a larger share of total surgical cost in LMIC settings than in U.S. tertiary centers, which means the time-saving advantages of SIT and ISBAS translate into proportionally larger relative cost-savings, even if absolute dollar figures are smaller. Third, where pediatric general anesthesia capacity is rate-limiting (e.g., a single pediatric anesthesia list per week), the ISBAS framework should be evaluated not only on per-encounter cost but also on aggregate program throughput, because each ISBAS encounter frees a future anesthesia slot for a different patient on the waiting list. Investigators reapplying the framework in any of these settings should report the four framework ingredients above transparently, deflate to a common local base year (see Section 4.5), and present results in local currency with parallel international-dollar conversion at purchasing-power-parity, where possible, to maximize cross-study comparability.

## 5. Conclusions

SIT had the shortest and least variable procedure time compared to all other pediatric angle surgeries while being at most negligibly more expensive. ISBAS shows a trend toward reduced anesthesia exposure time and costs compared to staged bilateral angle surgeries. Both Ahmed and Baerveldt tube shunting procedures were equivalent in cost and time. These findings may help pediatric glaucoma surgeons counsel families, select the optimal procedure for each patient, and responsibly steward healthcare resources.

## Figures and Tables

**Table 1:** Surgical intervention types with mean operative cost and mean total operating room time.

Procedure	N	N with EUA	Mean Operative Cost (Standard Deviation)	Mean OR Time (Minutes, Mean)
Goniotomy	18	14	$5750.60 (±1957.10)	119.4 (±47.5)
Harms trabeculotomy	16	8	$5596.01 (±1228.44)	123.8 (±40.1)
Single-Incision Trabeculotomy ab interno (SIT)	29	18	$7325.28 (±1249.11)	97.9 (±26.1)
iScience^TM^ trabeculotomy	12	6	$8599.97 (±2348.28)	172.2 (±52.2)
**Total Unilateral Angle Procedures**	**75**	**46**	**$6782.40 (±1952.03)**	**120 (±46.3)**
Immediate Sequential Bilateral Angle Surgery	15	11	$11,390.80 (±2396.26)	207.9 (±53.8)
Baerveldt with graft	35	14	$6845.08 (±923.32)	129.0 (±24.2)
Ahmed with graft	4	2	$6462.02 (±810.99)	120.0 (±27.4)
Shunt Revision	20	12	$4940.74 (±1397.41)	111.0 (±33.2)
Endoscopic Cyclophotocoagulation	10	5	$7469.07 (±1384.45)	124.8 (±32.5)
Diode trans-scleral cyclophotocoagulation	1	1	$4188.95 (±N/A)	90.0 (±N/A)
**All Unilateral Procedures**	**145**	**80**	**$6564.10**	**121.3**

**Table 2:** Total cost and total anesthesia time data for single-incision trabeculotomy compared to combined data for goniotomy and Harms trabeculotomy.

	Single-Incision Trabeculotomy	Goniotomy and Harms Trabeculotomy	*p*-Value
n	29	34	
cost (standard dev)	$7325.28 (±1249.11)	$5677.85 (±1632.55)	<0.0001
Total anesthesia time (standard dev)	97.90 (±26.13)	121.44 (±43.55)	0.0107

**Table 3:** Total cost and total anesthesia time data for single-incision trabeculotomy compared to the iScience microcatheter.

	Single-Incision Trabeculotomy	iScience Catheter	*p*-Value
n	29	12	
cost (standard dev)	$7325.28 (±1249.11)	$8599.97 (±2348.28)	0.0975
Total anesthesia time (standard dev)	97.90 (±26.13)	172.25 (±52.22)	0.0004

**Table 4:** Total cost and total anesthesia time data for single-incision trabeculotomy compared to all other unilateral angle procedures (goniotomy, Harms trabeculotomy, and iScience microcatheter).

	Single-Incision Trabeculotomy	Goniotomy, Harms Trabeculotomy, and iScience Microcatheter	*p*-Value
n	29	46	
cost (standard dev)	$7325.28 (±1249.11)	$6440.14 (±2232.82)	0.0312
Total anesthesia time (standard dev)	97.90 (±26.13)	134.70 (±50.66)	0.0001

**Table 5:** Operative cost and OR time for unilateral angle surgeries multiplied by a factor of 2 compared to ISBAS.

Variable	Unilateral Angle Surgery × 2	ISBAS ^a^	*p*-Value
n	75	15	
Cost ($)	$13,564.79 (±3904.05)	$11,390.80 (±2396.26)	0.0079
Time (min)	240.93 (±92.62)	207.93 (±53.76)	0.0684

**^a^** = Immediate sequential bilateral angle surgery.

**Table 6:** Baerveldt vs. Ahmed.

Variable	Baerveldt + Graft	Ahmed + Graft	*p*-Value
n	35	4	
Cost ($)	6845.08 (±923.32)	6462.02 (±810.99)	0.4284
Time (min)	129.00 (±24.19)	120.00 (±27.39)	0.567

**Table 7:** Baerveldt and Ahmed compared unilateral angle surgeries.

Variable	Angle	GDI (Ahmed + BV)	*p*-Value
n	75	39	
Cost ($)	6782.40 (±1952.02)	6805.79 (±910.26)	0.9307
Time (min)	120.47 (±46.31)	128.08 (±24.30)	0.2522

## Data Availability

The original contributions presented in this study are included in the article/Supplementary Material. Further inquiries can be directed to the corresponding author.

## Supplementary Materials

The following supporting information can be downloaded at https://www.mdpi.com/article/10.3390/jmahp14020034/s1. The following supporting information can be downloaded for this article: File S1, STROBE Checklist for cohort studies (completed for the present manuscript, with item-level, page-and-paragraph cross-references).

## Abbreviations

The following abbreviations are used in this manuscript:

SIT	Single-Incision ab Interno Trabeculotomy with the Trab360 [TM] or OMNI [TM] device
EUA	Exam Under Anesthesia
ISBAS	Immediate Sequential Bilateral Angle Surgery
OR	Operating Room
