# Supplementary material for: Operative Costs and Anesthesia Exposure Time for Pediatric Glaucoma Surgeries
Source: J Mark Access Health Policy. 2026 Jun 3;14(2):34. doi: 10.3390/jmahp14020034 (PMC13302323; doi:10.3390/jmahp14020034)
Supplement: Supplementary file 1 [file jmahp-14-00034-s001.zip › jmahp-3904243-supplementary.pdf]

Supplementary File S1.

STROBE Checklist for cohort studies (completed for the present manuscript, with item-level, page-and-paragraph cross-references).

Manuscript: *Operative Costs and Anesthesia Exposure Time for Pediatric Glaucoma Surgeries.*

This checklist follows the STROBE (Strengthening the Reporting of Observational Studies in Epidemiology) Statement for cohort studies (von Elm E, Altman DG, Egger M, et al. The Strengthening the Reporting of Observational Studies in Epidemiology (STROBE) Statement: Guidelines for reporting observational studies. Lancet 2007;370:1453–1457). For each item, we indicate where in the manuscript the corresponding information is reported. Section numbers refer to the revised manuscript with tracked changes accepted.

Section/Topic	Item	Recommendation	Reported on (Section / Paragraph)
Title and abstract			
Title and abstract	1 (a)	Indicate the study's design with a commonly used term in the title or the abstract.	Abstract, Methods sentence: "retrospective case series of consecutive pediatric glaucoma surgical encounters".
Title and abstract	1 (b)	Provide in the abstract an informative and balanced summary of what was done and what was found.	Abstract: revised Background, Objective, Methods, Results, and Conclusion paragraphs.
Introduction			
Introduction	2 — Background/rationale	Explain the scientific background and rationale for the investigation being reported.	Section 1, paragraphs 1–2 (techniques and FDA Drug Safety Communication on pediatric anesthesia neurodevelopment).
Introduction	3 — Objectives	State specific objectives, including any prespecified hypotheses.	Section 1, paragraph 3 (three prespecified comparisons and corresponding hypotheses).
Methods			
Methods	4 — Study design	Present key elements of study design early in the paper.	Section 2.1 Study Design and Reporting.
Methods	5 — Setting	Describe the setting, locations, and relevant dates, including periods of recruitment, exposure, follow-up, and data collection.	Section 2.2 Setting and Ethical Approval (UMMC, January 2012–August 2019).

Section/Topic	Item	Recommendation	Reported on (Section / Paragraph)
Methods	6 (a) — Participants	Cohort study: give the eligibility criteria, and the sources and methods of selection of participants.	Section 2.3 Participants; Section 2.4 Exclusion Criteria.
Methods	7 — Variables	Clearly define all outcomes, exposures, predictors, potential confounders, and effect modifiers.	Section 2.5 Outcome Variables and Cost Source.
Methods	8* — Data sources/measurement	For each variable of interest, give sources of data and details of methods of assessment (measurement). Describe comparability of assessment methods if there is more than one group.	Section 2.5 (cost source = institutional cost-accounting ledger; OR time = electronic anesthesia record wheels-in to wheels-out).
Methods	9 — Bias	Describe any efforts to address potential sources of bias.	Section 2.4 (exclusions); Section 4.5 Limitations (selection-by-surgeon bias and time-stamp error).
Methods	10 — Study size	Explain how the study size was arrived at.	Section 2.3 (consecutive enumeration of all eligible encounters in the study window; no a priori power calculation).
Methods	11 — Quantitative variables	Explain how quantitative variables were handled in the analyses. If applicable, describe which groupings were chosen and why.	Section 2.5 (cost and time as continuous variables); Section 2.6 Statistical Analysis.
Methods	12 (a) — Statistical methods	Describe all statistical methods, including those used to control for confounding.	Section 2.6 Statistical Analysis.
Methods	12 (b)	Describe any methods used to examine subgroups and interactions.	Section 2.6 (pairwise comparisons across procedure-type strata; ISBAS vs TSBAS subgroup).
Methods	12 (c)	Explain how missing data were addressed.	Section 2.7 Data Management (complete-case analysis; encounters with incomplete cost or time records were excluded — see Section 2.4).

Section/Topic	Item	Recommendation	Reported on (Section / Paragraph)
Methods	12 (d)	Cohort study: if applicable, explain how loss to follow-up was addressed.	Not applicable — the analytic outcome (cost, OR time) is captured intra-operatively; no follow-up was required.
Methods	12 (e)	Describe any sensitivity analyses.	Section 4.5 Limitations (sensitivity to inflation adjustment discussed); none formally executed in this retrospective dataset.
Results			
Results	13* (a) — Participants	Report numbers of individuals at each stage of study — eg numbers potentially eligible, examined for eligibility, confirmed eligible, included in the study, completing follow-up, and analysed.	Section 3.1 (160 surgical encounters analyzed); Section 2.4 (exclusion criteria).
Results	13 (b)	Give reasons for non-participation at each stage.	Section 2.4 Exclusion Criteria.
Results	13 (c)	Consider use of a flow diagram.	Cohort flow described narratively in Section 3.1; flow-diagram figure not included given the simplicity of the inclusion path.
Results	14* (a) — Descriptive data	Give characteristics of study participants (eg demographic, clinical, social) and information on exposures and potential confounders.	Table 1 (procedure mix and demographics); Section 3.1 narrative.
Results	14 (b)	Indicate number of participants with missing data for each variable of interest.	Section 2.4 and Section 3.1 (encounters with incomplete records excluded prior to analysis).
Results	14 (c) — Cohort study	Summarise follow-up time (eg average and total amount).	Not applicable — intra-operative outcome only.
Results	15* — Outcome data	Cohort study: report numbers of outcome events or summary measures over time.	Tables 2–4 (cost and OR time by procedure type, unilateral vs bilateral); Table 5 (broader classification).
Results	16 (a) — Main results	Give unadjusted estimates and, if applicable, confounder-	Tables 2–4 (means ± SD); pairwise p-values

Section/Topic	Item	Recommendation	Reported on (Section / Paragraph)
		adjusted estimates and their precision (eg, 95% confidence interval).	reported in Section 3.2–3.3.
Results	16 (b)	Report category boundaries when continuous variables were categorized.	Procedure-type categories enumerated in Tables 2–4; bilateral vs unilateral defined in Section 2.5.
Results	16 (c)	If relevant, consider translating estimates of relative risk into absolute risk for a meaningful time period.	Not applicable — outcome is continuous cost/time, not a risk.
Results	17 — Other analyses	Report other analyses done — eg analyses of subgroups and interactions, and sensitivity analyses.	Section 3.3 (ISBAS vs TSBAS subgroup); Section 3.4 Synthesis.
Discussion			
Discussion	18 — Key results	Summarise key results with reference to study objectives.	Section 4.1 Key Findings.
Discussion	19 — Limitations	Discuss limitations of the study, taking into account sources of potential bias or imprecision. Discuss both direction and magnitude of any potential bias.	Section 4.5 Limitations (selection bias, time-stamp error, no inflation adjustment, underpowered for rare adverse events and for the smallest continuous-outcome strata).
Discussion	20 — Interpretation	Give a cautious overall interpretation of results considering objectives, limitations, multiplicity of analyses, results from similar studies, and other relevant evidence.	Section 4.2 Interpretation in Context; Section 4.3 Mechanisms; Section 4.4 Clinical Implications.
Discussion	21 — Generalisability	Discuss the generalisability (external validity) of the study results.	Section 4.5 (single-center caveat); Section 4.6 International Generalizability of the Analytical Framework (European, Asian, LMIC reapplication).
Other information			
Other information	22 — Funding	Give the source of funding and the role of the funders for the present study and, if applicable,	Back matter: Funding statement.

Section/Topic	Item	Recommendation	Reported on (Section / Paragraph)
		for the original study on which the present article is based.	
**Give information separately for exposed and unexposed groups, where applicable.*			

— End of Supplementary File S1 —
